# Predictive Factors Influencing the Return to Sports Following Arthroscopic Knee Meniscectomy in Sports Persons: A Prospective Cohort Study

**DOI:** 10.7759/cureus.49334

**Published:** 2023-11-24

**Authors:** Anil Yadav, Sushmita Kushwaha, Rafat Kamal, Firoz A Khan, Aditya Sood

**Affiliations:** 1 Sports Medicine, Sports Injury Center, Safdarjung Hospital, New Delhi, IND; 2 Sports Medicine, Pandit Bhagwat Dayal Sharma Post Graduate Institute of Medical Sciences, Rohtak, IND

**Keywords:** arthroscopy, isokinetic strength, athletes, meniscectomy, return to sports

## Abstract

Meniscus tears are among the common knee injuries in sports, with arthroscopic meniscectomy being one of the most commonly performed orthopedic procedures. Return to sports of the same level following arthroscopic meniscectomy is an important aspect for athletes. Numerous factors may influence the time required for athletes to resume sports activities after meniscectomy. This prospective cohort study aimed to investigate the timeframe for returning to sports in athletes who underwent arthroscopic meniscectomy and to identify predictive factors that influence this return.

Ninety sports persons who had undergone arthroscopic meniscectomy were included in this study. The patients were analyzed for their time to return to sports and nine proposed predictive factors that may influence their return to sports. Out of the 90 participants, 75 were able to return to their previous activity level, while the remaining 15 were unable to do so. Among the nine pre-defined factors studied, age older than 25 years (p < 0.0001), participation in non-contact sports (p < 0.0001), and engagement in recreational activities (p < 0.0001) were found to be statistically significant.

In conclusion, this study reveals that with the increase in age, time to return to sports following arthroscopic meniscectomy increases. Additionally, athletes involved in non-contact sports and those having recreational sports activity levels experience greater delays in their return to sports as compared to athletes involved in combat and contact sports and athletes having elite and competitive sports levels, respectively.

## Introduction

Meniscus tears are a prevalent knee injury, with an approximate incidence of about 60-70 per 100,000 individuals [1-3]. Males are known to be more prone to meniscal injury than females with the male-to-female ratios ranging between 2.5:1 and 4:1 [1,4]. In cases of isolated meniscal injuries, medial meniscus tears are found to be more common than lateral meniscal tears [4,5]. On the other hand, in cases of anterior cruciate ligament tears, lateral meniscal injuries are more common in acute tears [2,6,7], whereas medial meniscus injuries are common in chronic anterior cruciate ligament insufficiency [2,5,6]. Isolated meniscal injuries typically result from a combination of axial loading and rotational forces, leading to shear stress on the meniscus [5]. The increasing participation in sports and recreational activities among the general population exposes more people to the risk of meniscal tears [2,8].

Meniscectomy is a frequently performed arthroscopic orthopedic procedure [1,3]. It can be partial, subtotal, or complete, and it is considered when repair is not feasible [8]. When making meniscectomy decisions, it is essential to consider patient's age, BMI, chondral lesions, the type and location of the tear, and the likelihood of healing for a favorable result [9,10]. During meniscectomy, it is advisable to preserve as much of the meniscus as possible [11-14].

Injured athletes often seek an early return to sports, as injuries can have detrimental effects on their careers. However, the time it takes for post-meniscectomy patients to return to sports remains uncertain. Factors such as age, gender, the side of the meniscus affected (medial or lateral), the type and location of the tear, condition of articular cartilage, the extent of meniscal resection, and the level of sports activity influence the duration of the return to sports participation following meniscectomy [15-18]. The objective of our study is to investigate the timeline for a return to sports in post-meniscectomy patients and identify the factors that impact their ability to resume sports activities after meniscectomy.

## Materials and methods

This prospective cohort study was conducted at the Sports Injury Centre, VMMC and Safdarjung Hospital in New Delhi from 2018 to 2021. Ethical approval for the study was taken from the Institutional Ethics Committee of VMMC and Safdarjung Hospital prior to the start of the study (S.no. IEC/VMMC/SJH/Thesis/October-2016). The study comprised 90 athletic patients with meniscal tears in a stable knee, who were clinically and radiologically evaluated and scheduled for arthroscopic meniscectomy. Patients with any knee ligament injuries or a history of previous knee surgery were excluded from the study, and written informed consent was obtained from each participant.

Prior to the surgery, data related to the participant’s medical history, including age, gender, the type of sports played, time since the injury, side of the injury, mechanism of injury, activity level before the injury, history of locking, or previous arthroscopic surgery, were documented. This was followed by the documentation of clinical and radiological evaluation findings. Intra-operative findings during the arthroscopic meniscectomy were also recorded. Post-operatively, patients underwent rehabilitation and were followed up according to the planned rehabilitation protocol (Table 1).

**Table 1:** Post-operative rehabilitation and follow-up protocol.

Phase- 1 (0-1 week)	
Goals	Exercise program
Control swelling, maintain knee extension and knee flexion to 90^0^	Passive knee extension, heel slide, full weight bearing, gentle range of motion and prone knee bending/ hanging
Phase- 2 (1-2 weeks)	
Goals	Exercise program
Eliminate swelling and full range of motion	Range of motion drills, standing calf stretching, Hamstring stretch on wall, isometric Vastus medialis oblique/ hip abduction/ hip adductor exercise and straight leg raises
Phase- 3 (2-3 weeks)	
Goals	Exercise program
Full squat (double limb) and static proprioception training	Resisted knee extension, step-up, wall squat with a ball, static cycling and gait re-education exercises
Phase- 4 (3-5 weeks)	
Goals	Exercise program
Full strength, endurance of affected limb and sports specific drills	Neuromuscular proprioceptive exercises, resisted knee extension/ flexion, resisted leg abduction/ adduction and sports specific exercises
Return to Sports	
Permitted, if the patient has no swelling, no joint line tenderness, perform painless hop test and has less than 15% deficits of Quadriceps and Hamstring power by isokinetic strength analyzer.	

Statistical analysis

The data was entered in Excel spreadsheets (Microsoft Office Excel 2016; Microsoft Inc., Redmonds, WA) and analysis was done using licensed IBM SPSS Statistics for Windows, Version 21 (Released 2012; IBM Corp., Armonk, New York, United States). Categorical variables were expressed in numbers and percentages (%), while continuous variables were presented as mean ± standard deviation and median. Normality of the data was assessed using the Kolmogorov-Smirnov test. Quantitative variables were compared using unpaired t-tests, while qualitative variables were compared using the Chi-Square test. Logistic regression analyses were conducted to identify significant factors influencing the return to sports in post-meniscectomy athletes. Odds ratios with 95% confidence intervals were calculated for the pre-defined variables (predictive factors), and their significance was assessed. A p-value of <0.05 was considered statistically significant.

## Results

The observations made in this study were based on prospective evaluation of 90 athletic patients with meniscal tears planned for arthroscopic meniscectomy. The descriptive statistics of the population under study is shown in Table 2.

**Table 2:** Sample characteristics of the population.

S.no.	Variable	Type of variable and data
1.	Time since injury(weeks)	Continuous Variable
	Median	15.5
	Inter-quartile range	10-35
2.	Age	Categorical Variable
	<25 years	56 (62.22%)
	25-35 years	28 (31. 11%)
	>35years	6 (6.67%)
3.	Gender	Categorical Variable
	Male	80 (88.89%)
	Female	10 (11.11%)
4.	Type of sports	Categorical Variable
	Combat	56 (62.22%)
	Contact	22 (24.44%)
	Non-contact	12 (13.33%)
5.	Chondral Changes	Categorical Variable
	Present	6 (6.67%)
	Absent	84 (93.33%)
6.	Side of meniscal tear	Categorical Variable
	Medial meniscus	50 (55.56%)
	Lateral meniscus	40 (44.44%)
7.	Site of meniscal tear	Categorical Variable
	Anterior horn	7 (7.78%)
	Body	48 (53.33%)
	Posterior horn	35 (38.89%)
8.	Type of meniscal tear	Categorical Variable
	Longitudinal	5 (5.56%)
	Radial	10 (11.11%)
	Horizontal	7 (7.78%)
	Complex	46 (51.11%)
	Bucket handle	21 (23.33%)
	Flap	1 (1.11%)
9.	Amount of meniscal resection	Categorical Variable
	Small	66 (73.33%)
	Medium	20 (22.22%)
	Large	4 (4.44%)
10.	Activity level before injury	Categorical Variable
	Elite	16 (17.78%)
	Competitive	22 (24.44%)
	Recreational	52 (57.78%)

Factors predicting return to the same activity level post-arthroscopic meniscectomy

It was observed that out of 90 post-arthroscopic meniscectomy patients, 75 (83.33%) patients were able to return to the same activity level as before the injury, while 15 (16.67%) were not. To determine whether these predictive factors influence the return to the same activity levels post-meniscectomy, logistic regression was done. Univariate logistic regression of all the factors, with their regression coefficients, standard error, p-value, odds ratio, and limits of 95% confidence interval (C.I.) for odds ratio was calculated. A p-value 0f <0.05 at 95% C.I. was considered to be significant for a given factor. Age categories of more than 25 years were found to be statistically significant and the remaining other factors were found to be insignificant (Table 3).

**Table 3:** Univariate logistic regression for return to same activity level. B: Regression coefficient; S.E: standard error; C.I.: confidence interval

Factors	B	S.E.	p-value	Odds ratio	95% C.I. for odds ratio	
					Lower	Upper
Age						
<25 years (reference)	---	---	---	1	---	---
25-35 years	-2.861	.817	.0005	.057	.012	.284
>35years	-2.603	1.126	.021	.074	.008	.674
Gender						
Male (reference)	---	---	---	1.000	---	---
Female	-.887	.758	.242	.412	.093	1.818
Type of sports						
Combat (reference)	---	----	----	1	---	---
Contact	-.552	1.215	.650	.576	.053	6.233
Non-contact	-.989	1.097	.367	.372	.043	3.195
Chondral Changes	.000	1.134	1.000	1.000	.108	9.229
Side of meniscal tear						
Medial meniscus (reference)	---	----	---	1.000	---	---
Lateral meniscus	.429	.568	.450	1.536	.505	4.673
Site of meniscal tear						
Anterior horn (reference)	---	---	---	1	---	---
Body	-.024	1.155	.983	.976	.101	9.389
Posterior horn	-.405	1.160	.727	.667	.069	6.474
Amount of meniscal resection						
Small (reference)	---	---	---	1	---	---
Medium	.125	.708	.860	1.133	.283	4.539
Large	-.511	1.201	.671	.600	.057	6.316
Activity level before injury						
Elite (reference)	---	---	---	1	---	---
Competitive	.357	1.059	.736	1.429	.179	11.384
Recreational	-.630	.829	.447	.532	.105	2.702

**Figure 1:**
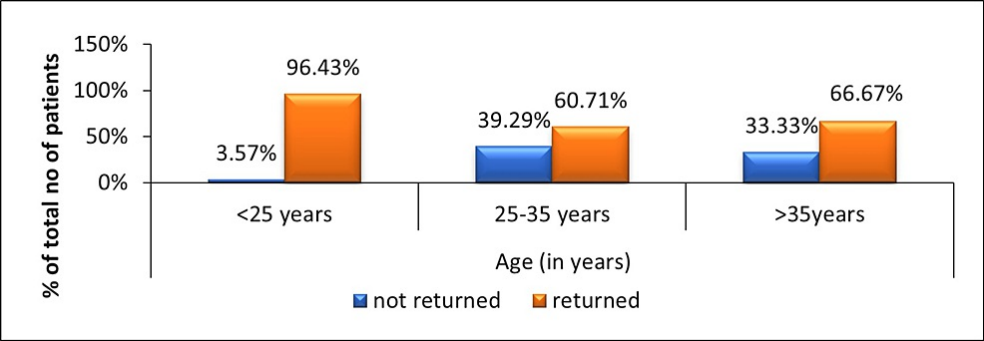
Frequency of patients who returned to the same activity level with respect to the age category.

The results show that age as a factor predicts return to the same activity level post-meniscectomy. Figure 1 shows the correlation between predictive factor age with respect to return to the same activity level. It shows that patients in the age group of <25 years have a maximum rate of return to the same activity level 96%, while in the age group 25-35 years, they have 60% and >35years they have 66%.

Factors predicting the time taken to return to sports post-arthroscopic meniscectomy

**Figure 2:**
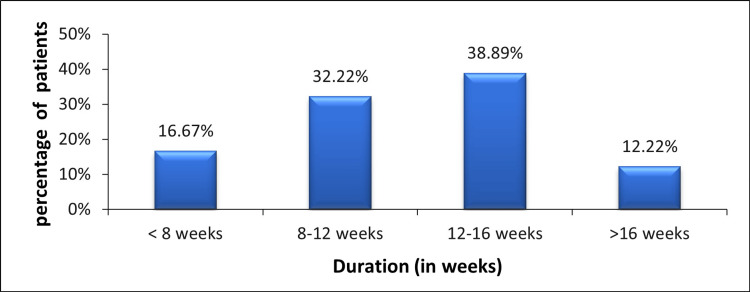
Percentage of patients returned to sports with respect to duration.

Figure 2 shows the percentage of patients who returned to sports with respect to duration. It shows that the majority of patients (87.78%) of the population have returned to sports within 16 weeks following arthroscopic meniscectomy.

To determine whether these pre-defined factors predict the time taken to return to play, logistic regression was done. Univariate linear regression of factors for the time to return to sports with their regression coefficients, standard error, p-value, odds ratio, and limits of 95% C.I. for odds ratio was calculated. It was observed that only age (p<0.0001), type of sports (p<0.0001), and activity level (p<0.0001) were found to be related to the time taken to return to sports post-meniscectomy (Table 4).

**Table 4:** Univariate linear regression for the time taken to return to play post-meniscectomy. B: Regression coefficient; S.E.: standard error

Factors	Unstandardized Coefficients		Standardized Coefficients	p-value	95.0% Confidence Interval for B	
	B	S.E.	Beta		Lower Bound	Upper Bound
Age						
<25 years	Taken as reference					
25-35 years	23.625	4.481	.503	< .0001	14.711	32.539
>35years	16.818	4.253	.455	< .0002	8.312	25.325
Gender						
Male	Taken as reference					
Female	-3.475	7.850	-.047	.659	-19.075	12.125
Type of sports						
Combat	Taken as reference					
Contact	4.061	5.445	.131	.461	-7.030	15.151
Non-contact	18.185	2.600	.652	< .0001	12.993	23.376
Chondral Changes	17.012	9.734	.183	.084	-2.332	36.356
Side of meniscal tear						
Medial meniscus	Taken as reference					
Lateral meniscus	-9.470	4.867	-.203	.055	-19.142	.202
Site of meniscal tear						
Anterior horn	11.839	9.466	.169	.217	-7.148	30.826
Body	Taken as reference					
Posterior horn	5.386	4.817	.174	.270	-4.350	15.121
Type of meniscal tear						
Longitudinal	Taken as reference					
Radial	-11.100	14.836	-.203	.468	-43.151	20.951
Horizontal	-3.457	7.711	-.140	.663	-20.637	13.723
Complex	-2.081	3.736	-.079	.580	-9.589	5.427
bucket handle	-2.324	2.951	-.159	.439	-8.413	3.766
Flap	-5.040	6.416	-.366	.476	-22.855	12.775
Amount of meniscal resection						
Small	Taken as reference					
Medium	-11.521	5.975	-.206	.057	-23.403	.361
Large	-3.061	5.850	-.063	.603	-14.734	8.613
Activity level before injury						
Elite	Taken as reference					
Competitive	3.455	2.859	.197	.235	-2.343	9.253
Recreational	21.587	1.669	.847	< .0001>	18.255	24.918

**Figure 3:**
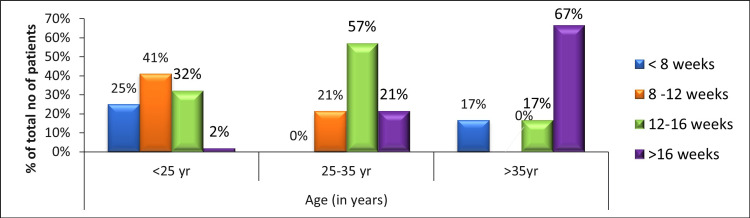
Frequency of time taken to return to play with respect to age categories.

This study observed that age >25 years, non-contact sports, and recreational activity level predicts the time to return to sports in a post-meniscectomy sportsperson. Figure 3 shows the frequency of time taken to return to play with respect to age categories in post-meniscectomy patients. It shows that as age increases, the time to return to sports following arthroscopic meniscectomy increases.

**Figure 4:**
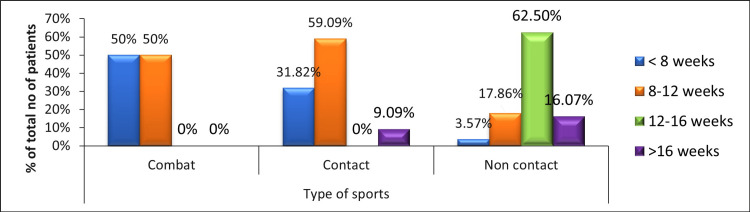
Frequency of time taken to return to play with respect to the type of sports played.

Figure 4 shows the frequency of time taken to return to play with respect to the type of sports played following arthroscopic meniscectomy. It shows that the time to return to play is higher for non-contact sports (like cricket) as compared to combat (like boxing) and contact sports (like soccer).

**Figure 5:**
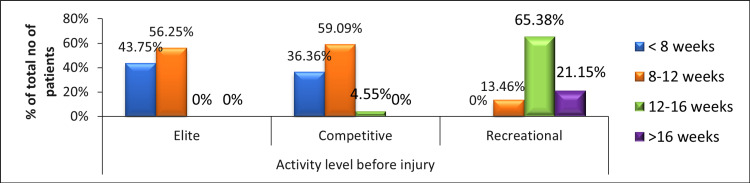
Frequency of time taken to return to play with respect to the activity level before injury in post-meniscectomy patients.

Figure 5 depicts the frequency of time taken to return to play with respect to the activity level before injury in post-meniscectomy patients. It shows that the time taken to return to sports is higher for patients who had a recreational activity level before injury as compared to elite and competitive level athletic patients.

## Discussion

Meniscectomy is among the most commonly performed orthopedic procedures. However, time to return to sports after meniscectomy is not uniform and depends on a number of factors. These factors may influence the time to return to sports in post-meniscectomy sportspeople. These factors are age, sex, cartilage status, injured meniscus (medial or lateral), type of meniscal tear, site of meniscal tear, amount of resection, and sports activity level before injury.

We have performed the study on 90 patients with meniscal tears visiting our department who planned for arthroscopic meniscectomy. These predictive factors were enquired before surgery and intra-operative meniscal and cartilage damage was documented. Post-operatively, all patients were put on the standard protocol of the rehabilitation program. The program was individualized depending on the progress of patients. All the observations were analyzed at the end of the study.

The result of this study showed that among these pre-defined factors, some factors do influence or predict the time taken to return to sports and the maximum level of activity that can be performed after surgery.

A similar study on 56 athletes found that the young age group (<30 years) returned to sports earlier than the older age group (>30 years), the mean period for return to sports was 54 days in the young age group and was 89 days in old age group (p = 0.0013) [15]. By contrast, another study on 77 lateral meniscectomy athletic patients found that age at the time of surgery was not significantly different between those who did and did not return to play [16]. In the present study, age is found to significantly affect the time to return to sports after meniscectomy. In the higher age group, the time taken to return to play is more in contrast to younger patients (<25 years= 73 days, 25-35 years= 97 days, >35 years= 107 days) (p<0.0001).

There is no study available on whether the type of sports influences the time to return to sports in post-meniscectomy athletes. This study found that the type of sports significantly affects the time to return to sports with sports people of combat (59 days) and contact (63 days) sports returning to sports earlier than non-contact sports (100 days) (p<0.0001).

The present study found significant differences in time to return to play between elite/competitive and recreational (p< 0.0001) groups. The mean period of return was 57 days in the elite group, 60 days in the competitive group, and 100 days in the recreation group. The findings favored a similar study that reported a significant difference among activity levels (p = 0.0036) between the recreation group and elite and competition groups [15]. The mean period was 54 days in the elite group, 53 days in the competition group, and 88 days in the recreation group.

Other pre-defined factors like gender, cartilage damage, side, site and type of meniscal tear, and amount of meniscal resection had mixed literature with respect to return to sports [15,17-20]. In this study, factors like gender, cartilage damage, side, site and type of meniscal tear, and amount of meniscal resection during meniscectomy do not predict return to sports following arthroscopic meniscectomy.

Additionally, we also studied the percentage of patients who returned to the same activity level and the influences of various factors on it. We found that out of a total of 90 patients, 75 patients i.e. 83.33% were able to return to the same activity level whereas 15 (16.67%) patients were not able to return to the same activity level. Among these factors, only age is significantly related to the patient’s return to the same activity level (p=0.005).

Our study has its own limitations. Firstly, we have not taken into consideration the psychological factors which are known to play a crucial role in players' return to sports following injury. Secondly, the study population was mostly recreational sports persons, who therefore had to return to a relatively low level of sports activity.

## Conclusions

The findings of this prospective cohort study shed valuable light on the predictive factors influencing the return to sports following arthroscopic knee meniscectomy in sports persons. The study examined a cohort of individuals who underwent this common surgical procedure and identified several key factors like age, type of sports, and activity level before injury that play a crucial role in determining the likelihood of successfully returning to their sports activities. These findings underline the multifactorial nature of returning to sports after arthroscopic knee meniscectomy. It is not solely dependent on the surgical procedure but is influenced by age, the type of sports involved, the pre-injury activity level, etc.

Understanding these factors can aid healthcare professionals in developing more tailored rehabilitation and recovery plans for sports persons, ultimately helping them achieve their goal of returning to their chosen athletic endeavors. Further research in this area can continue to refine our understanding of the complex interplay between these predictive factors and provide better guidance for athletes and surgeons in optimizing post-operative outcomes.
